# Comprehensive Statistical Analysis of Skiers’ Trajectories: Turning Points, Minimum Distances, and the Fundamental Diagram

**DOI:** 10.3390/s25051379

**Published:** 2025-02-24

**Authors:** Buchuan Zhang, Andreas Schadschneider

**Affiliations:** Institute for Theoretical Physics, University of Cologne, 50937 Köln, Germany

**Keywords:** skier traffic, trajectory, turning point, minimum distance, fundamental diagram, safety

## Abstract

In recent years, an increasing number of accidents at ski resorts have raised significant safety concerns. To address these issues, it is essential to understand skiing traffic and the underlying dynamics. We collected 225 trajectories, which were analyzed after a correction process. To obtain a quantitative classification of typical trajectories we focus on three main quantities: turning points, minimum distance, and the fundamental diagram. Our objective was to analyze these trajectories in depth and identify key statistical properties. Our findings indicate that three factors—turning angle, curvature, and velocity change—can be used to accurately identify turning points and classify skiers’ movement styles. We found that aggressive skiers tend to exhibit larger and less stable turning angles, while conservative skiers demonstrate a more controlled style, characterized by smaller, more stable turns. This is consistent with observations made for the distribution of the minimum distance to other skiers. Furthermore, we have derived a fundamental diagram which is an important characteristic of any traffic system. It is found share more similarities with the fundamental diagram of ant trails than those of highway traffic.

## 1. Introduction

Skiing is a popular winter sport that combines recreational enjoyment with complex physical and dynamical processes. Understanding the statistical properties of skiers’ trajectories is crucial for improving techniques, enhancing safety, and optimizing ski resort design. This investigation focuses on three key quantities: turning points, minimum distance between skiers, and the fundamental diagram relating density, speed, and flow in collective motion.

Previous studies have explored various aspects of skiing dynamics. Reid et al. [1] analyzed ski motion characteristics in slalom, providing insights into ski trajectories and their implications for technique and tactics. Similarly, Gilgien et al. [2] determined the center of mass kinematics in alpine skiing using differential GPS, offering precise data for studying skiing dynamics. Supej [3] introduced differential specific mechanical energy as a quality parameter in racing alpine skiing, enhancing the understanding of energy behavior during turns.

Turning techniques significantly influence performance and safety. Müller et al. [4] compared ski turn techniques of experienced and intermediate skiers, highlighting the effects of skill level on turning characteristics. Sandbakk et al. [5] examined downhill turn techniques and physical characteristics in cross-country skiing, emphasizing technique and fitness. Antekolovic et al. [6] investigated the relationship between speed through gates and body geometry in elite skiers.

In terms of biomechanics and kinematics, Vaverka et al. [7] developed a method for kinetic analysis of ski turns based on ground reaction forces, enabling detailed examination of forces during different turn phases. Hirano and Tada [8,9] conducted numerical simulations of turning skis, showing the effect of ski side-cut on turn radius. Kondo et al. [10] and Hirose and Doki [11] utilized inertial sensors for motion analysis, enhancing understanding of skier movements. Thorwartl et al. [12] validated a sensor-based ski deflection measurement, demonstrating potential for smart ski equipment.

Safety and collision avoidance are essential, with minimum distance between skiers being a critical factor. Ruedl et al. [13] investigated causes of collisions on ski slopes, providing insights into factors that increase collision risks. Matter et al. [14] analyzed skiing accidents over 15 years, identifying injury patterns and emphasizing the importance of equipment and behavior. Lochner et al. [15] reviewed fatal skiing accidents, highlighting the need for improved safety measures. Additionally, Ruedl et al. [16] examined fatigue’s impact on ACL injuries, highlighting the importance of physical conditioning. Yamazaki et al. [17] investigated severe head injuries in alpine skiing, aiding helmet design.

Modeling and simulation of skiing dynamics have been approached using various methods. Treiber et al. [18] introduced a model for cross-country ski marathons based on models for highway traffic. Holleczek and Tröster [19] developed a particle-based model for ski slope traffic. Korecki et al. [20] adapted the social force model for downhill skiing simulation. Fu et al. [21] studied turning curvature’s effect on flow dynamics, relevant to skier density. He et al. [22] modeled collision avoidance in vehicles, principles applicable to skiing. Cai and Yao [23] optimized ski trajectories using control theory. Eberle et al. [24] conducted a multibody simulation study of ski vibrations caused by slope roughness, essential for improving slope design and skier comfort. Combinido and Lim [25] modeled U-turn traffic flow, which can inform simulations of skier interactions during turns. Li and Sun [26] investigated vehicle lane-changing dynamics and its effects, offering insights applicable to skier movements during lane changes on slopes.

Advancements in technology have facilitated detailed data collection and analysis of skier trajectories. Gilgien et al. [2] provided accurate kinematic measurements using GPS. Boltes and Seyfried [27,28] developed methods for trajectory extraction from videos, applicable to skiing. Zhang et al. [29] simulated skiing areas using cellular automata. Delibasic et al. [30] analyzed skier trajectories using RFID data. Yurtsever et al. [31] proposed methods using GPS data for trajectory history. Holst and Jonasson [32] applied machine learning techniques to classify skiing movements, focusing specifically on cross-country skiing techniques, or “gears”. The method was evaluated on data from elite skiers during a training race, highlighting its applicability to professional athletic analysis. Instead of emphasizing internal body dynamics, we aim to model external skier motion, including trajectory patterns and skier interactions, to better understand the dynamics of skiing on slopes.

Gao et al. [33] use wearable technology for large-scale field data collection aligning with projects focusing on real-world activity monitoring. In contrast, our approach primarily relies on software to directly collect data from video footage. While their focus is mainly on the physical properties of the skiers’ bodies, our research emphasizes the traffic properties of skiers as they glide down the slope.

Despite the extensive research, gaps remain in the detailed analysis of turning points, quantification of minimum distances between skiers, and the construction of the fundamental diagram for skier movement. This study aims to fill these gaps through statistical analysis of skiers’ trajectories, integrating advanced modeling techniques and empirical data. By enhancing understanding in these areas, we seek to improve safety measures, optimize skiing techniques, and contribute to the design of better ski resorts.

The structure of this paper is as follows: In Section 2 we summarize the data collection and processing. In Section 3, turning points and their properties are analyzed. Section 4 focuses on the minimum distance between skiers and other agents, examining how this distance changes over time. In Section 5, we present fundamental diagrams to explore the relationships between density, flow, and velocity. Finally, Section 6 provides a discussion and conclusions based on our findings.

## 2. Data Collection and Processing

As basis for our analysis, we use video footage provided by the KFV (Kuratorium für Verkehrssicherheit, Board for Safety in Traffic), Vienna (Austria). The data were collected at different slopes in the downhill skiing resort Grosseck-Speiereck in Austria. A single camera with fixed position was used. The trajectories of individual skiers have been extracted from the videos using the software package PeTrack [28].

Since information about the type of camera, its exact position, distance to the slope, etc., was missing we have developed a method for data correction that allows one to incorporate the effects of perspective, etc. Details can be found in [34], where a representative example of an original trajectory versus a corrected one is shown in Figure 6. These corrections mainly affect absolute values, whereas the shape of distributions is not much changed. In the following, we use the corrected data for further analysis.

## 3. Turning Points

**Figure 1:**
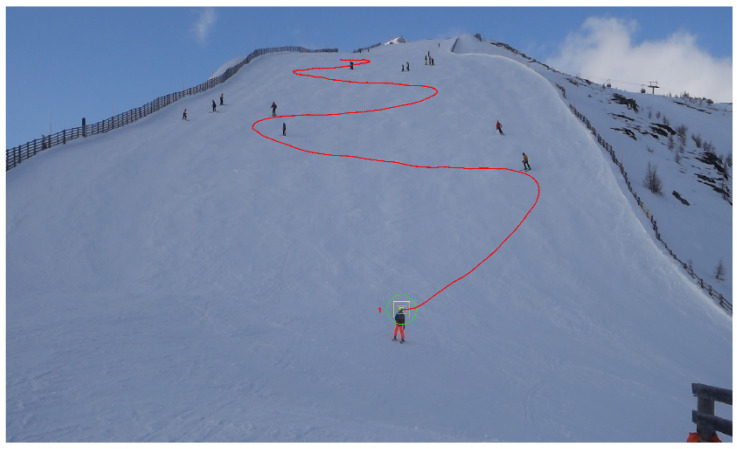
A typical trajectory of a skier, where the red line represents the skier’s movement path, and the number “1” indicates its identification by the software. A total of five turning points can be identified along the trajectory.

In our analysis of skier trajectories, we have observed that turning points play a significant role in shaping the overall path. This section will focus on a detailed study of these turning points. Typically, a turning point is defined as the moment when a skier changes direction, either to the right or left, from their initial trajectory. Such directional changes are often clearly visible, even from a considerable distance, as demonstrated by the example in Figure 1.

### 3.1. Factors Associated with Turning Points

We identify three key factors that influence the turning points: the turning angle, the change in velocity, and the curvature. Below, we provide their definitions and the methods for calculating each.

The turning angle describes the amount of change in the direction of a skier’s path at a given point. In simple terms, it represents the skier’s change in direction depending on the position. Suppose a skier’s trajectory is represented by a series of coordinates $P_{1}\left(x_{1},y_{1}\right)$ and $P_{2}\left(x_{2},y_{2}\right)$ in a two-dimensional plane. The turning angle between these two points can be calculated using the angle between two adjacent vectors.

(1) $$\theta =\cos^{-1}\left(\frac{\vec{v_{1}}\cdot \vec{v_{2}}}{|\vec{v_{1}}\left|\right|\vec{v_{2}|}}\right)\;.$$

Speed refers to how fast the skier is moving over a given period, typically measured in meters per second (m/s). The change in speed describes how the skier’s velocity increases or decreases during their movement. It is determined from the skier’s velocity $v_{1}=\left|\vec{v_{1}}\right|$ at time $t_{1}$ and $v_{2}=\left|\vec{v_{2}}\right|$ at time $t_{2}$:

(2) $$∆v=\frac{v_{2}-v_{1}}{t_{2}-t_{1}}$$

To calculate the instantaneous speed along the trajectory, the derivative of the position with respect to time is used:

(3) $$v\left(t\right)=\sqrt{{\left(\frac{dx}{dt}\right)}^{2}+{\left(\frac{dy}{dt}\right)}^{2}}$$

where x(t) and y(t) represent the skier’s position as functions of time.

Curvature measures how sharply the trajectory curves at a given point, indicating the rate of change in direction. A higher curvature means a sharper turn, while a lower curvature indicates a gentler curve. For a trajectory in a plane, the curvature *k* at a point (x(t),y(t)) of the trajectory is given by

(4) $$k=\frac{|\dot{x}\ddot{y}-\dot{y}\ddot{x}|}{{\left({\dot{x}}^{2}+{\dot{y}}^{2}\right)}^{3/2}}$$

where $\dot{x}$ and $\dot{y}$ are the first derivatives of the trajectory with respect to *t*, representing the velocity components. $\ddot{x}$ and $\ddot{y}$ are the second derivatives of the trajectory, representing the acceleration components.

The local maxima in the curvature graph correspond to key turning points in the skier’s trajectory, indicating sharp directional changes. These peaks likely represent moments of intense maneuvering or changes in terrain.

**Figure 2:**
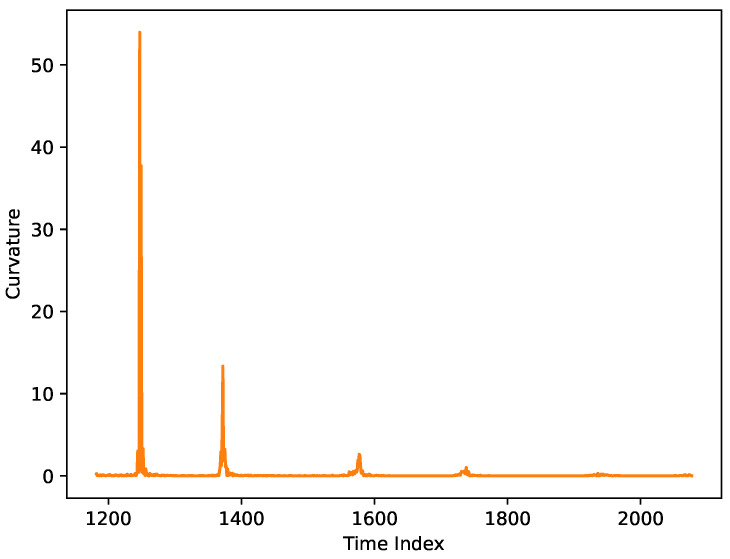
Curvature along a typical trajectory.

As shown in Figure 2, which represents the curvature plot of the original trajectory (Figure 1), several distinct curvature peaks are observed, with the highest exceeding 50, indicating a particularly abrupt turn. In the time interval [1200, 1400], multiple sharp turns are evident, suggesting that the skier executed several rapid maneuvers during this period. After time 1400, the curvature stabilizes, reflecting smoother and more consistent skiing with fewer directional changes. Overall, apart from these pronounced peaks, the skier’s trajectory appears relatively stable, indicating smoother movement throughout most of the course.

### 3.2. Identifying Turning Points by Turning Angles

**Figure 3:**
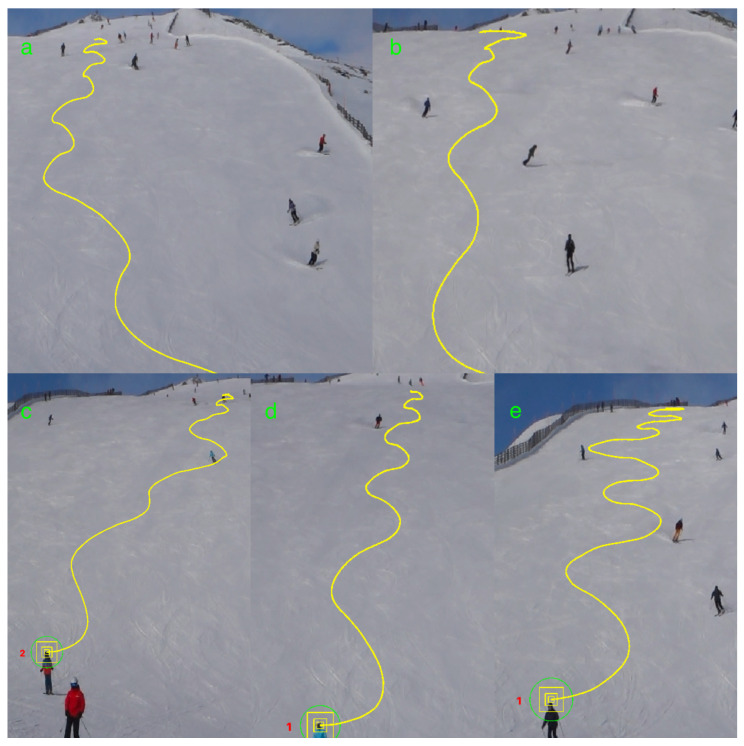
Displayed are the uncorrected trajectories of five skiers, with (**a**–**e**) denoting individual skiers. The yellow lines trace their respective paths, and the numbers correspond to their software-detected identifiers.

**Figure 4:**
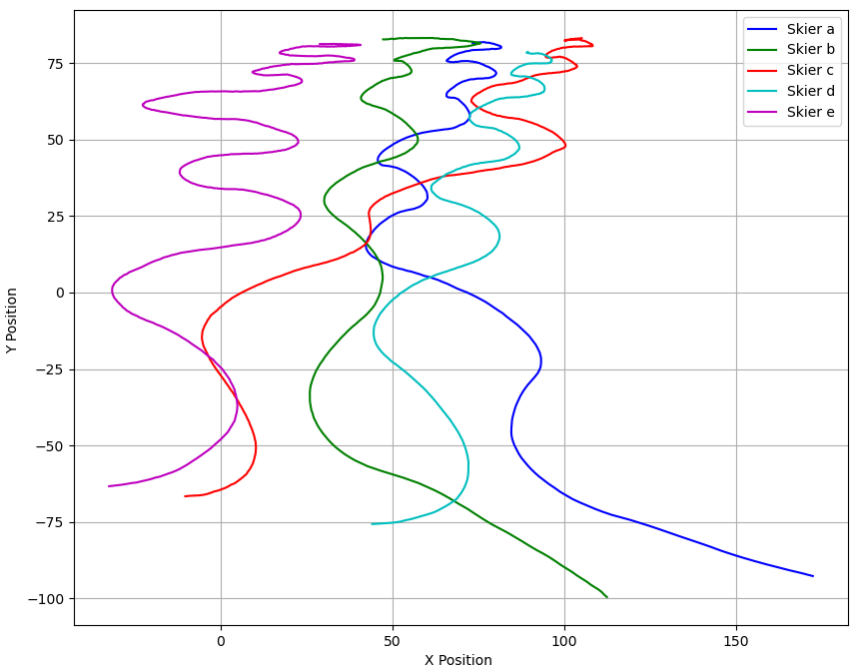
Corrected trajectories used for further analysis.

We present five distinct trajectories (Figure 3), each illustrating the unique skiing styles of different skiers. The data were collected using PeTrack [28]. To ensure accurate analysis, we applied a correction process to the extracted trajectories. The primary adjustments involved perspective correction, spatial scaling, and positional alignment. While the pre-processed and post-processed figures look visually similar, the corrected trajectory more accurately reflects the skier’s motion by compensating for camera-induced distortion. These corrected data, shown in Figure 4, allowed us to analyze each skier’s curvature, turning points, overall velocity, and velocities in both the x and y directions. Our primary objective is to provide insights for operators to better understand the dynamics of skiing and its associated properties which allows them to improve safety and comfort in skiing areas.

In this section, we introduce the fundamental changes in angle during the skiers’ movement on the slope, which are crucial for identifying turning points. Any turn made by a skier is accompanied by a change in the direction of motion. Therefore, we analyzed the directional changes along their trajectories to better understand these turning points.

**Figure 5:**
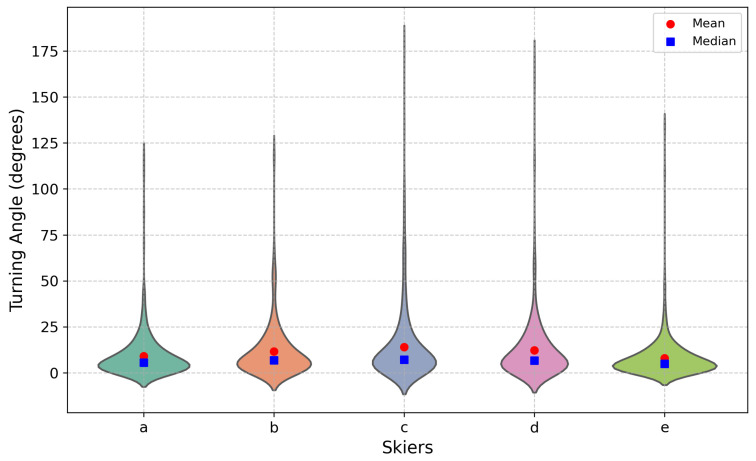
The turning angle varies along the trajectory, with (a–e) representing five individual skiers, respectively.

The violin plot (see Figure 5) illustrates the distribution of turning angles for each skier (A to E). Skier A’s turning angles are concentrated in the lower range (0°–25°), with only a few larger turns reaching up to 100°. Skier B shows a similar distribution, though slightly more dispersed, with angles extending up to 50°. Skiers C and D display broader distributions, frequently reaching angles of 150° or more. Notably, both skiers have a considerable difference between their mean and median values compared with others, indicating significant variability and more dramatic changes in turning angles. Skier E exhibits the narrowest distribution, with most turns concentrated below 25°, reflecting a focus on smaller, more controlled turns.

The results indicate that different skiing styles can be distinguished, in agreement with those of Section 3.1. In the following, we try to confirm this by a more detailed analysis of the curvature of the trajectories.

### 3.3. Analysis and Classification of Skiers’ Trajectories Curvature

To identify the turning points of each skier’s trajectory, we can use the calculated curvature and its changes along the trajectory.

**Figure 6:**
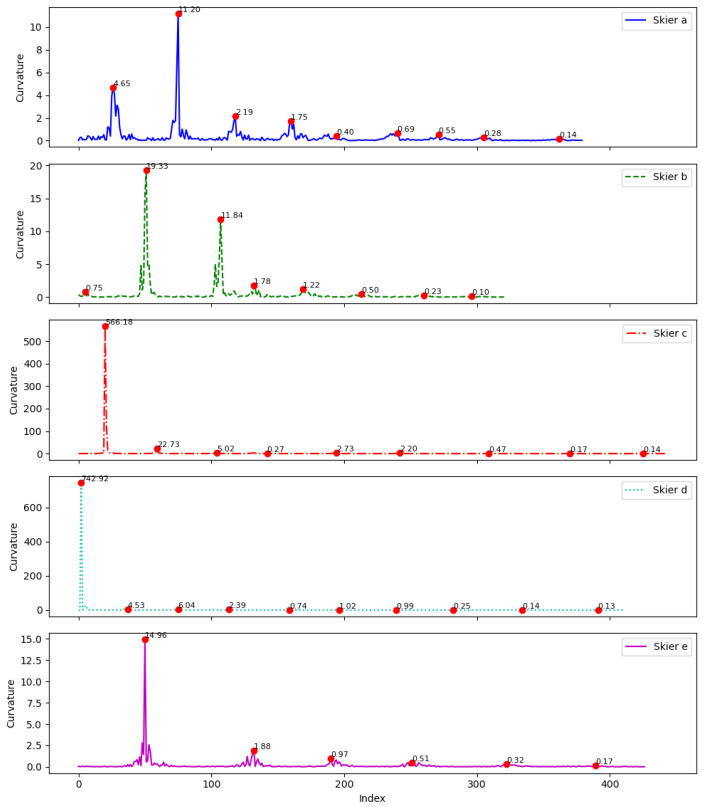
Curvature of the skier’s trajectory, with red dots indicating turning points.

As seen in Figure 6, the curvature method effectively identifies the turning points for each skier. The curvature graph for Skier A shows frequent turning points with relatively small curvature values (e.g., 4.65, 11.20, 2.19, 1.75), with most values below or close to 10, indicating a generally smoother trajectory with occasional gentle turns. Skier B’s graph exhibits several more pronounced peaks (e.g., 19.33, 11.84, 1.78), suggesting a more winding path characterized by frequent directional changes. Skier C displays a sharp peak at 566.18, followed by smaller peaks (e.g., 72.73, 5.02), indicating significant and unstable changes in direction. Similarly, Skier D shows a sharp peak at 742.92, followed by smaller peaks (e.g., 4.53, 6.04), reflecting large variations in curvature. Skier E’s graph reveals a relatively stable progression in turning angles, with only one small peak at 14.96, followed by smaller peaks (e.g., 1.98, 0.97, 0.51), indicating consistent and smooth turns throughout the journey.

In summary, Skiers A and E primarily exhibit smaller turns, indicative of smoother skiing with longer turn radii. In contrast, Skiers C and D demonstrate a combination of sharp and smaller turns, with significant variation between turns, reflecting a more aggressive skiing style. Skier B represents an intermediate level between the two, characterized by larger turns followed by smaller ones.

### 3.4. Relationship Between Velocity Changes and Turning Points

Velocity changes can also serve as indicators of turning points. During a turn, skiers often decelerate or accelerate, which can be identified by examining the rate of change of velocity over time.

**Figure 7:**
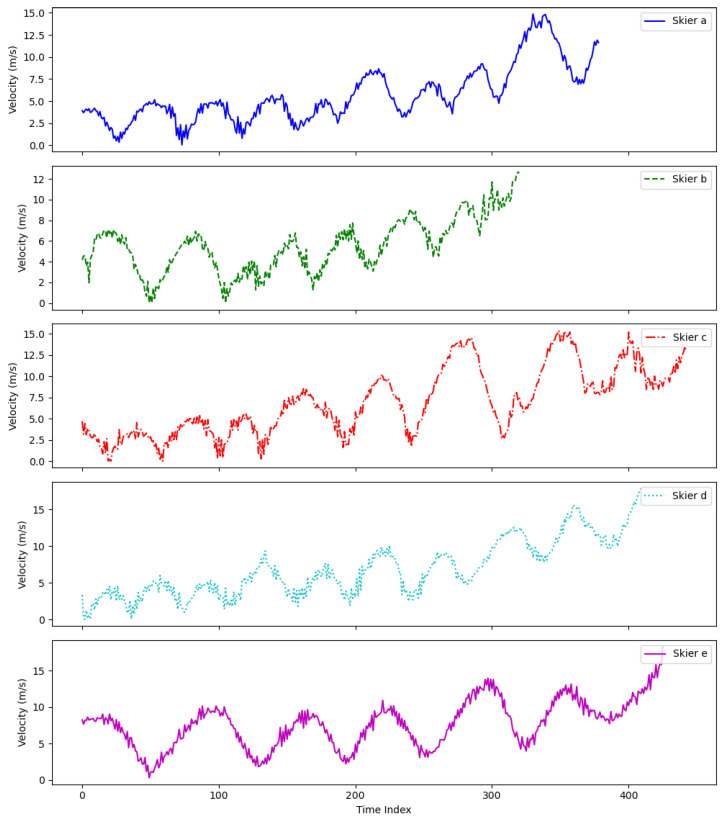
Plot of five skiers’ velocity variation along the trajectory.

As observed in Figure 7, the velocity continuously varies throughout the trajectory, reflecting the skiers’ adjustments in response to turns and changes in the path. Skier A’s velocity graph shows a stable wave pattern, with velocities primarily ranging between 0 m/s and 5 m/s, and rarely exceeding 7 m/s. This suggests a conservative approach, with limited fluctuations in speed. Similarly, Skier B’s velocity remains stable, predominantly below 7 m/s, though it occasionally ranges between 6 m/s and 10 m/s. In contrast, Skier C’s velocity graph exhibits pronounced fluctuations, with values ranging from 0 m/s to 15 m/s, indicating a wider range of speeds and reflecting sharp turns and frequent velocity changes. Skier D displays a similar pattern to Skier C but with smaller amplitude, suggesting adjustments following sharp turns. Skier E’s graph shows periodic fluctuations, indicating a consistent pattern of acceleration and deceleration with each turn.

Overall, Skiers C and D demonstrate more aggressive skiing behavior, characterized by frequent and significant velocity changes. In contrast, Skiers A and E adopt a more conservative style, with smaller and more stable variations in speed. Skier B presents an intermediate approach, with generally stable velocity changes but occasional bursts of higher speed.

**Figure 8:**
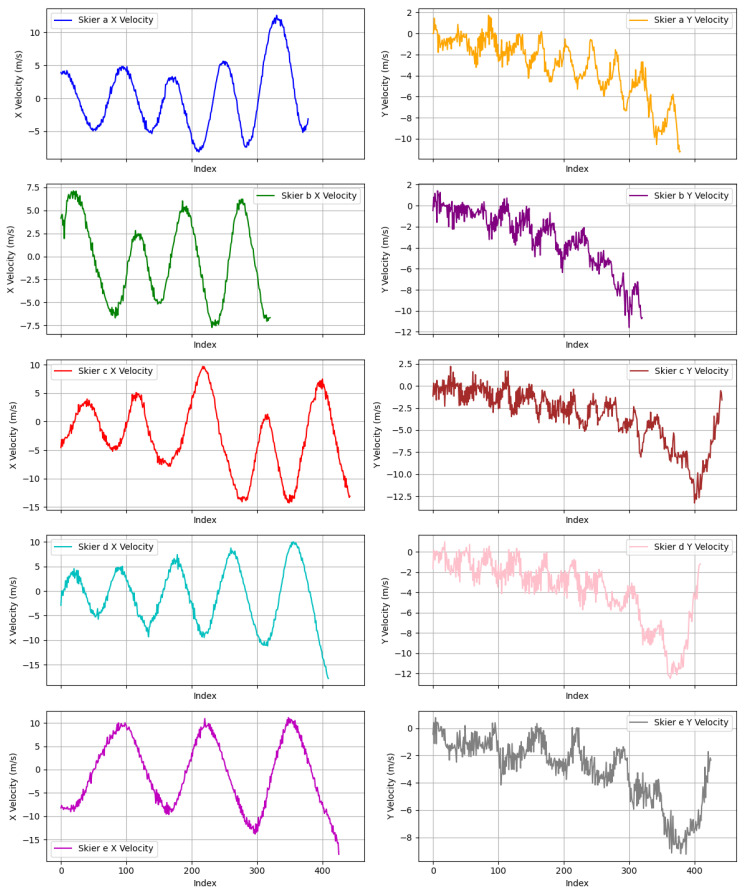
The changes in velocity of five skiers along their x-direction and y-direction.

We also calculated the changes in velocity for the five skiers along both the x- and y-directions, as shown in Figure 8. It is widely understood that a turning point is typically indicated by the x-velocity reaching zero. Additionally, we observe that the y-velocity does not reach zero at every turn, instead gradually increasing as the skier approaches the bottom of the slope. By analyzing these velocity changes, we can clearly identify the number of turning points in each of the five trajectories. This approach proves to be an effective method for detecting turning points, thereby enhancing our understanding of the skiers’ movement patterns.

**Figure 9:**
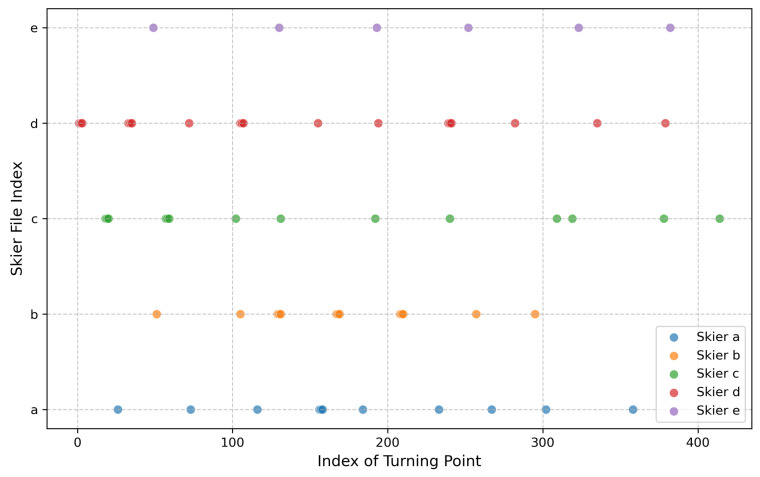
Scatter plot of the turning points of five skiers. The x-coordinate represents time measured in frames in the video.

Finally, based on the previous analysis of velocity changes in the x- and y-directions, we can determine the exact locations of the turning points. For details, refer to Figure 9.

**Figure 10:**
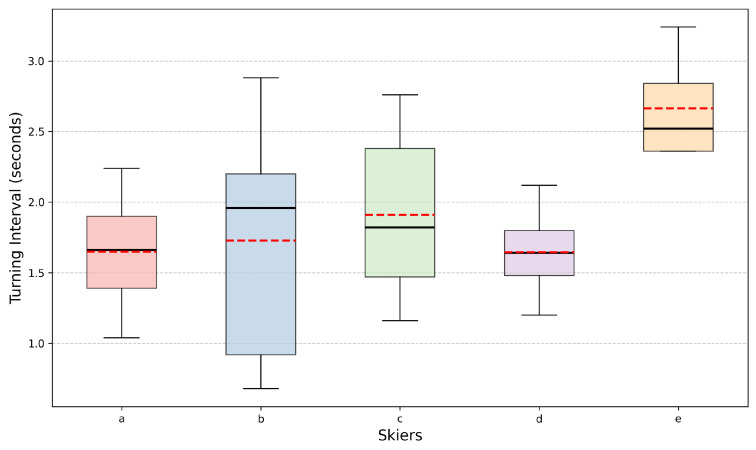
Turning intervals of the skiers, with the red dashed line denoting the value of the Mean Turning Interval and the black solid line indicating the value of the Median Turning Interval.

We also observe that skiers exhibit certain patterns in their turning intervals. As shown in Figure 10, skiers A, B, C, and D have mean turning intervals around 1.75 s, with skier E being the exception at 2.66 s. Additionally, the median turning intervals for these skiers are close to their respective mean values, suggesting consistency in their turning behavior. From this, we can conclude that most skiers make turns in less than 2 s on average.

### 3.5. Summary

In this section, we introduced three key factors (turning angle, velocity change, and curvature) that are closely related to the shape of the trajectories and can be used to identify turning points. We then applied this framework to five individual trajectories to demonstrate the process of identifying and classifying turning points. The analysis reveals that at each turning point, the turning angle, curvature, and velocity change vary significantly between skiers. This analysis enables us to infer different skiing styles: for instance, aggressive skiers tend to exhibit sharp turns at higher velocities, while more stable skiers are characterized by lower velocities and smaller turns. This approach offers valuable insights into the dynamics of skiers’ trajectories and can help operators better understand their customers, ultimately improving the overall skiing experience.

## 4. Minimum Distance to the Other Agents

So far, we have considered only the motion of a single skier to understand the basic properties of their trajectories. As we are interested in the traffic properties of downhill skiing, it is essential to highlight that the most critical aspect is their safety during movement. This necessitates considering their proximity to other skiers, particularly whether they might encounter unfamiliar individuals or lose control and come too close to another skier, potentially leading to minor collisions or other accidents—scenarios we aim to avoid. Therefore, we will focus on the concept of minimum distance to better understand this behavior in skiing dynamics, especially the interactions between skiers. Additionally, we would like to clarify that, for simplicity, the term “minimum distance” will sometimes be abbreviated as “MD” throughout the text.

### 4.1. Data Collection

**Figure 11:**
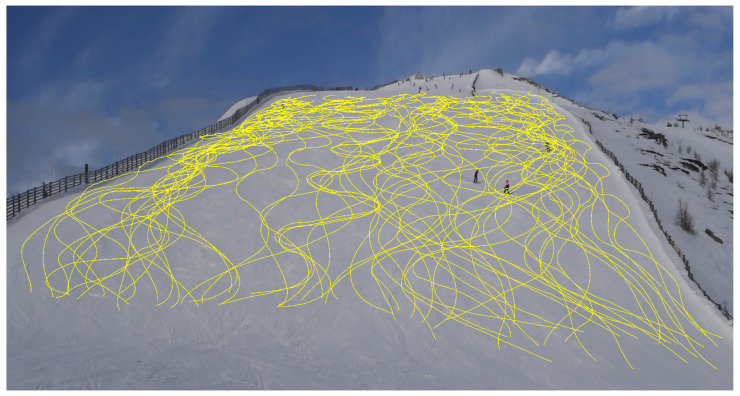
60 original trajectories from the slope.

To identify fundamental patterns, 60 trajectories (Figure 11) have been extracted from a randomly selected segment of the video. To ensure the reliability of the data, all skiers present in the segment were considered in the analysis. It is important to highlight that each of these trajectories represents an individual skier, with none belonging to any predefined groups or pairs. Furthermore, these sixty skiers were tracked over a continuous period of approximately 172 s, offering a comprehensive dataset for further analysis. In the selection, trajectories that are not continuous, e.g., because skiers stopped at some point, have been excluded from the analysis.

**Figure 12:**
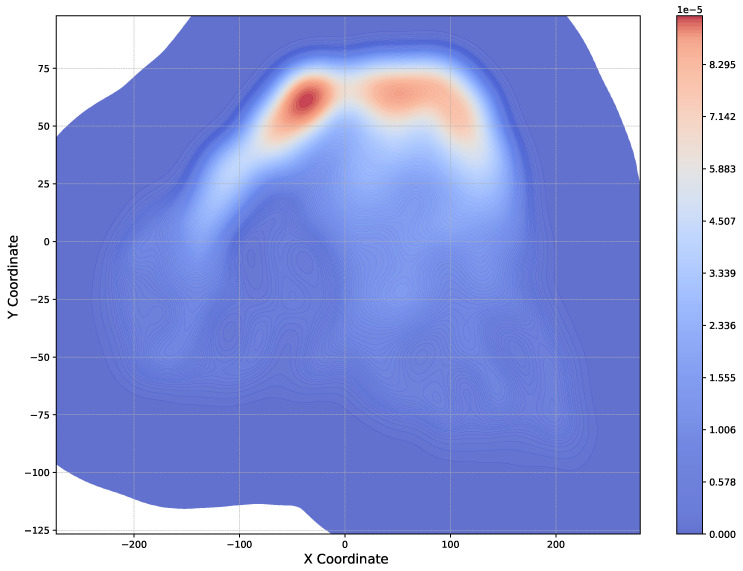
Heatmap for the positions of the skiers.

Having obtained the trajectories of skiers on the slope, we have generated a heatmap, as shown in Figure 12. This heatmap allows to visualize areas which are attractive and frequented by many skiers. These attractive areas usually correspond to higher densities and are particularly concerning because they increase the likelihood of safety risks, such as collisions, falls, or even a chain reaction of falls. Therefore, it is essential for operators to identify these high-risk zones where such incidents are more likely to happen, enabling them to take preventive measures to enhance skier safety.

From Figure 12 we observe that skiers are densely distributed in the top part of the piste, which provides an indication of higher probabilities for collisions or other accidents in this area. Near the bottom of the piste, the heatmap appears predominantly blue, indicating lower skier density. This suggests that particular caution is needed at the beginning of the slope, as it is identified as an accident-prone area.

### 4.2. Minimum Distance Matrix

To provide valuable insights, we aim to identify clusters of skiers who typically move within their comfort zones. This means they maintain a minimum distance from other agents, allowing them to move freely without risking close encounters. Based on this principle, we will analyze the minimum distance between agents to understand the dynamics of skier movement and identify potential risk zones. The minimum distance between any two skiers A and B at time t is computed using the Euclidean distance:

$$d_{AB}\left(t\right)=\sqrt{{\left(x_{A}-x_{B}\right)}^{2}+{\left(y_{A}-y_{B}\right)}^{2}}$$

**Figure 13:**
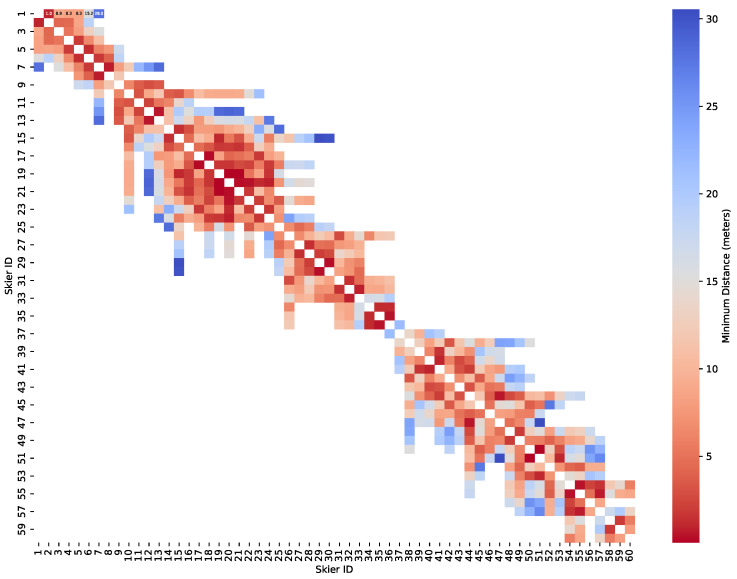
Matrix for minimum distance between skiers.

This process is repeated for all skiers at each frame, allowing us to construct a matrix representing the minimum distances between skiers over time. Figure 13 illustrates a heatmap of these distances.

In this section, we construct a matrix that represents the minimum distance between each pair of skiers as they move on the slope. We will then explain the process used to derive this matrix. The starting point is the available data for each skier, which includes their frame numbers along with their corresponding x- and y-coordinates. The first step involves identifying the common frames between any two skiers by finding the intersection of their respective frame data. Next, we initialize the minimum distance between the two skiers to infinity (inf) to ensure that any subsequently calculated distance will be smaller than this initial value. The script then iterates through all the common frames, calculating the Euclidean distance between the two skiers for each frame. If a calculated distance is found to be smaller than the current minimum distance, the script updates the minimum distance accordingly. This process continues until all common frames have been evaluated, resulting in a matrix of minimum distances between all pairs of skiers.

In the matrix presented in Figure 13, we observe a color gradient representing the varying distances between skiers, ranging from cold blue to warm red. Blue indicates that skiers are relatively far apart, while red signifies that they are in close proximity. Notably, certain clusters of skiers are in very close proximity to one another, particularly the group of skiers numbered 17 to 24, with distances between them typically ranging from 1 to 2 m. This suggests that these skiers may be following similar movement patterns and operating within the same skiing area.

Another notable observation is that most of the red squares, indicating closer proximity, are concentrated along the diagonal line of the matrix. This pattern suggests that these skiers are often neighbors or skiing in close proximity to one another. However, it is also evident that not all neighboring skiers are very close. For instance, skiers 36 and 37, despite being adjacent in the matrix, maintain a considerable distance of nearly 20 m between them, as indicated by the color gradient.

In contrast, the blue squares, which represent skiers who are farther apart, tend to appear between skiers with significantly different numbers. This could be attributed to the skiers being on different parts of the slope at different times. For example, skiers numbered 15 and 30 have a distance of nearly 30 m between them, suggesting that one skier may be near the bottom of the slope while the other remains near the top. This indicates that their paths likely did not cross closely during the observation period.

### 4.3. Minimum Distance Change over Time

Building on the general findings presented earlier, we now focus on a more granular analysis of the minimum distances between skiers. To achieve this, we have constructed a figure that tracks each skier’s minimum distance to other skiers over time. This visualization enables us to precisely monitor how skiers approach or distance themselves from one another throughout their descent.

**Figure 14:**
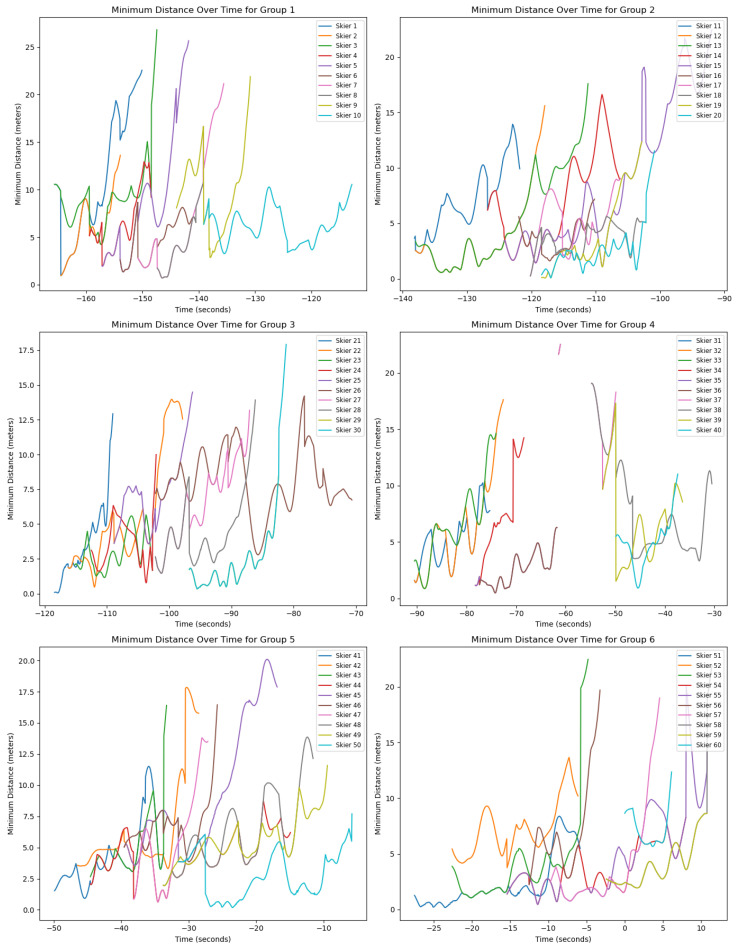
Minimum distance to other agents. For more clarity, we have divided the sixty skiers into 6 groups which are here shown separately.

As illustrated in Figure 14, we observe that the minimum distances between skiers fluctuate over time, with values rising and falling intermittently. Despite these variations, there is a discernible upward trend in the minimum distances over the course of the descent. This pattern may be attributed to the increase in velocity as skiers progress downhill; as velocity increases, skiers generally maintain greater distances from one another to avoid collisions, which would otherwise be inevitable at higher velocities.

Notably, the analysis reveals instances where the minimum distance between certain skiers approaches zero, suggesting near-miss situations where collisions were narrowly avoided. Moreover, the line chart displays several overlapping trajectories, indicating moments when specific pairs of skiers were closest to each other simultaneously.

**Figure 15:**
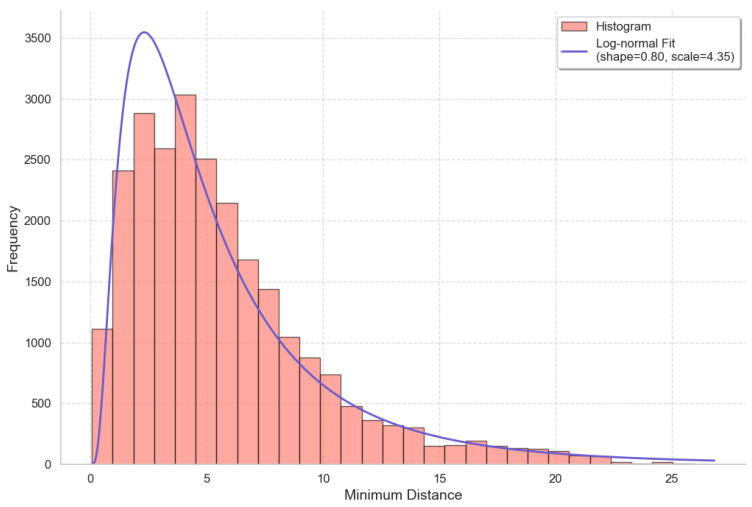
Distribution of minimum distances across all skiers and frames with a log-normal fitting function.

To further examine how closely skiers approach one another, we turn to Figure 15, which provides a detailed view of the distribution of minimum distances. This figure reveals the primary values of the minimum distances between skiers. It is evident that the majority of these minimum distances cluster around the 3–5 m range. As the minimum distance increases beyond this range, its frequency declines sharply, indicating that most skiers rarely maintain substantial distances from their neighbors while skiing.

### 4.4. Minimum Distance Attribute for Each Skier

To gain a more comprehensive understanding, we present a detailed chart containing specific metrics for each skier throughout their journey on the slope. This chart includes the minimum distance observed, the mean minimum distance, and the standard deviation of these minimum distances, offering a nuanced view of skiers’ behavior. This information is summarized in Table 1. In our analysis, we define any distance less than 2 m as a “close contact incident”, highlighting moments of heightened collision risk.

**Figure 16:**
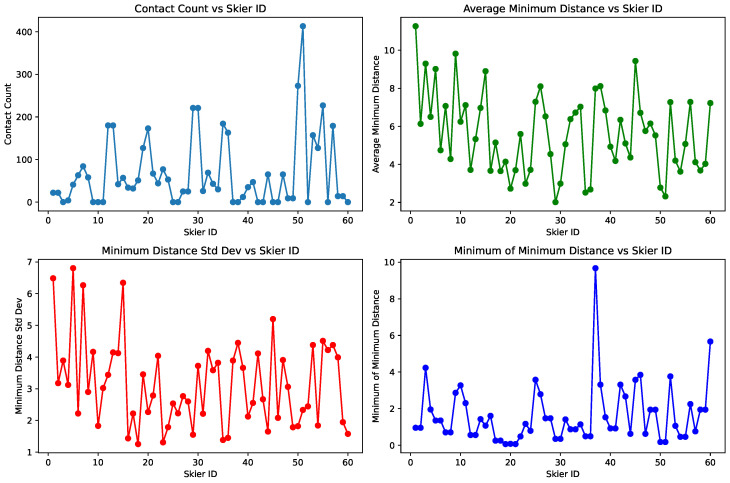
Statistical analysis of Contact count, Average MD, MD standard deviation, Minimum MD.

As observed from the table, skiers often share similar values with their neighbors, particularly in metrics such as contact count and minimum MD. To further analyze their behavioral patterns, we have generated a line chart (see Figure 16) that illustrates the trends between different skiers.

From the contact count, we can see that skiers near number 50 have the highest values, suggesting that these skiers may engage in more aggressive movements, frequently coming into close proximity with others. In contrast, skiers like near numbers 10 and 37 are much more conservative, as they show almost no contact with other skiers.

Looking at the average MD, there is no dramatic variation between skiers, with most values falling within the range of 2 to 8 m. Only a few skiers exhibit a much larger average MD, indicating that most skiers maintain a moderate distance from others throughout their descent.

When we examine the standard deviation of the MD, skiers around number 7 stand out with significantly higher variability compared to others, indicating that their movement is more flexible and unstable. Conversely, skiers like near number 24 and 35 have much lower MD standard deviation, suggesting they maintain a stable distance throughout their journey.

Lastly, focusing on the minimum MD values, skiers near number 20 come very close to others, with minimum distances approaching zero. In contrast, skiers near number 37 have exceptionally high minimum MD values, indicating that they remained far away from others during their entire run. For most skiers, however, the minimum MD ranges from 1 to 4 m, implying that only a few skiers take the risk of getting very close to others, with the majority opting for a safer distance to maintain while skiing.

**Figure 17:**
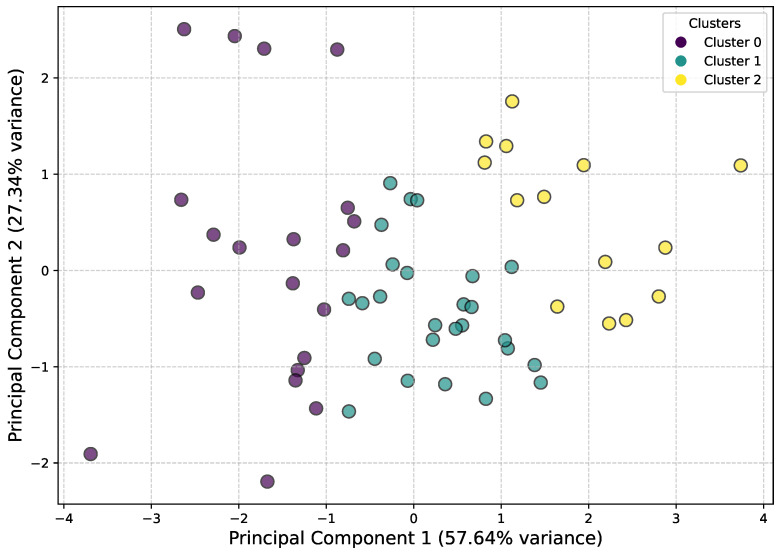
KMeans Clustering with PCA (2D projection).

To analyze the characteristics of the skiers, we need to first perform a cluster analysis (shown in Figure 17) and then look at the means of each category of skiers on four features (number of contacts, average minimum contact distance, standard deviation of MD, minimum MD). By comparing the mean differences of each category, we can summarize the unique characteristics of each category of skiers.

As shown in Table 2, the three clusters demonstrate clear distinctions in their characteristics. Cluster 0 has the lowest contact count, averaging 14.4, suggesting that skiers in this group are more conservative in their movements, maintaining greater distances from others. In contrast, Cluster 1 has a contact count of 36.58, slightly higher than Cluster 0, while Cluster 2 stands out with a significantly higher contact count of 201.79, indicating much more frequent close contact with other skiers.

In terms of average minimum distance (MD), Cluster 0 exhibits the largest value at 7.95, indicating a preference for maintaining greater distance from others. Cluster 1 follows with an average MD of 4.97, and Cluster 2 has the smallest average MD of 3.44. The similarity between Clusters 1 and 2 suggests that skiers in these groups tend to ski closer to others compared to those in Cluster 0.

Regarding stability, Cluster 0 has a higher variability, with a standard deviation of 4.07, reflecting less stability in their movements. In comparison, Cluster 1 (2.58) and Cluster 2 (2.9) show similar and much lower variability, indicating more stable movement patterns.

For the minimum MD, we observe that Cluster 0 has the largest value at 2.97, while Cluster 1 has a value of 1.25, and Cluster 2 the smallest at 0.43. This highlights a distinct difference in skiing styles across the clusters. Skiers in Cluster 0 tend to maintain greater distance from others, while those in Cluster 2 are much closer in proximity. Cluster 1 represents a more moderate behavior in this aspect.

In summary, Cluster 0 skiers avoid contact with others, maintain larger distances, but exhibit less stability in their skiing patterns. Cluster 1 skiers represent a middle ground, with moderate contact frequency, medium proximity to others, and stable movements. Cluster 2 skiers have the highest frequency of close contact, ski in close proximity to others, but remain highly stable, suggesting a higher skill level compared to the other groups.

Based on these observations, it is natural to assume that the skill levels of the clusters can be ranked as follows: Cluster 2 has the highest skill level, Cluster 1 occupies the middle, and Cluster 0 represents the least skilled group.

**Figure 18:**
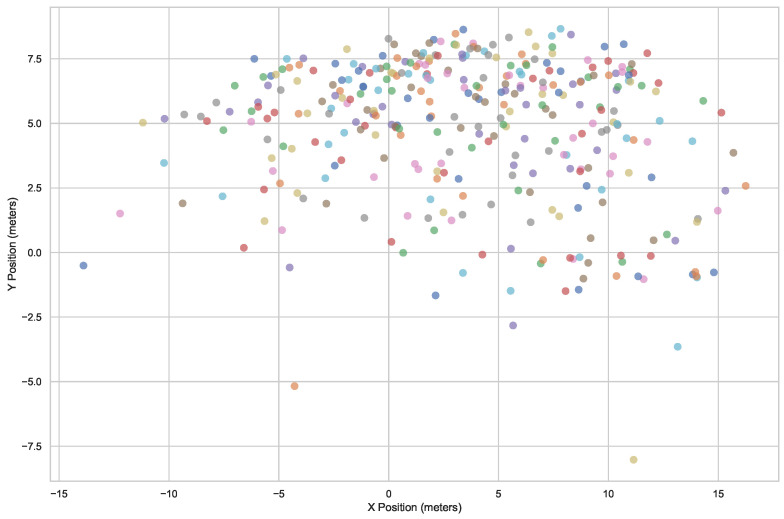
The closest contact event between each pair of skiers, where points represent the positions of the skiers at the moment when they were closest to each other.

We have also generated a plot that shows where skiers experience close contact incidents on the slope, as seen in Figure 18. From this figure, it is evident that the majority of these incidents occur in the upper part of the slope. This could be attributed to the fact that the skiers start at a similar position before using the full width of the slope. At the top of the hill, the available area for skiing is more limited, leading to higher skier density and, consequently, an increased likelihood of close contact situations. Additionally, skiers near the top of the hill tend to have lower velocities. Another contributing factor is that this area often serves as a transition point to other paths or slopes, where many skiers pause, either waiting or resting. This crowding effect likely increases the risk of contact between skiers as they navigate the narrower spaces near the top of the slope.

### 4.5. Summary

In this section, we conducted a comprehensive study on the minimum distance between skiers, a critical factor in understanding skier interactions and safety risks. Initially, we extracted 60 trajectories from the footage and performed a preliminary analysis of their spatial distribution, revealing that certain areas appeared more crowded than others.

We then focused on studying the minimum distance (MD) between skiers, which plays a significant role in understanding their behavior on the slope. By examining the minimum distance matrix, we could infer each skier’s closest proximity to others throughout the entire process and identify their nearest neighbors.

Next, we analyzed how the MD changes over time, allowing us to observe how skiers approach or move away from others during their descent. Following this, we investigated the distribution of MD values, which showed that the majority of skiers maintain distances between 3 to 5 m from others.

To further understand proximity, we set a threshold of 2 m to identify close-contact incidents and created a table with four key characteristics to compare the skiers. Additionally, we performed a cluster analysis, grouping skiers into three categories based on their behaviors.

Finally, we generated a map plot illustrating the locations where the closest contact incidents occurred. This visualization provides valuable insights for operators to identify high-risk areas and improve safety measures, ensuring both the safety and comfort of the skiers.

In this section, we analyzed the minimum distance (MD) between skiers to evaluate interaction patterns and safety risks. Our findings show that most skiers maintain an MD of 3–5 m, with close-contact incidents (MD < 2 m) occurring infrequently but concentrated in specific high-risk areas. Similar to pedestrian interactions in constrained spaces [35], skiers adjust spacing dynamically based on density. Cluster analysis revealed three skier behavior groups: those maintaining stable, safe distances; those with inconsistent proximity patterns; and a high-risk group prone to close contacts. By tracking MD changes over time, we identified dynamic behaviors where skiers approach or retreat from others during descent. A spatial distribution analysis highlighted crowded zones where close-contact incidents are most frequent, offering critical insights for safety interventions. These results emphasize the need for targeted management of high-risk areas to enhance skier safety and slope efficiency.

While this study primarily focuses on absolute minimum distances between skiers, an important factor that could further enhance our analysis is the consideration of relative movement directions. Skiers moving in the same direction at similar speeds may be able to maintain smaller minimum distances safely, while those on converging trajectories may require greater separation to avoid collisions. Future research could incorporate relative velocity angles into the modeling of skier interactions, allowing for a more dynamic classification of risk zones on ski slopes. This approach could provide more refined safety metrics and improve the accuracy of skier traffic simulations.

## 5. Fundamental Diagrams

Up to now, we have investigated the turning points, and minimum distances of skiers. Now, we aim to extend this analysis by examining an important aspect of any traffic systems, the fundamental diagram. The fundamental diagram is crucial in understanding the collective properties of skier movement and is widely used in traffic flow theory.

For this analysis, we have selected a specific time period from the recorded footage to examine the skiers’ density, velocity, and flow, as well as the relation between these variables. Since skiers moving down a slope exhibit behaviors similar to traffic phenomena, it is essential to analyze their movement from a traffic flow perspective, utilizing relevant theories.

**Figure 19:**
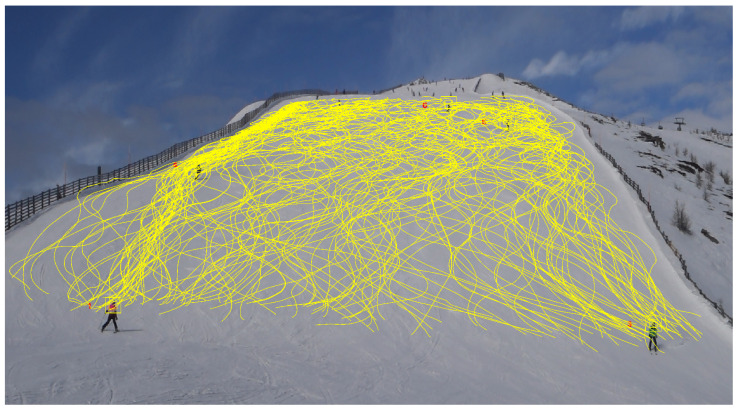
Sections of the slope along which skiers glide, with each line representing an individual skier’s trajectory.

We collected approximately 160 skiers’ trajectories from the footage (Figure 19) before proceeding with the data analysis. The total time span of the recorded footage is 374 s. To calculate skier density and flow, we used the mean distance between an individual and their five nearest neighbors to represent local density. Flow is defined as the product of density and velocity. Below, we outline the specific process used to calculate these values.

### 5.1. Calculation of Density and Flow

To estimate the density, we first calculate the distance between each skier and other skiers using the Euclidean distance. For two skiers with coordinates ${\vec{r}}_{i}=\left(x_{i},y_{i}\right)$ and ${\vec{r}}_{j}=\left(x_{j},y_{j}\right)$, the distance $d_{ij}$ between them is given by

(5) $$d_{ij}=\sqrt{{\left(x_{j}-x_{i}\right)}^{2}+{\left(y_{j}-y_{i}\right)}^{2}}\;.$$

The K-Nearest Neighbors (KNN) algorithm is used to find the nearest *k* skiers around each skier. Here, k=5, meaning we compute the distance to the 5 nearest neighbors for each skier.

After finding the nearest *k* neighbors, we calculate the average distance to these neighbors. Let $d_{i1},d_{i2},\ldots ,d_{ik}$ represent the distances between skier $p_{i}$ and their nearest *k* neighbors. The average distance $\bar{d_{i}}$ is given by

(6) $$\bar{d_{i}}=\frac{1}{k}\sum_{j=1}^{k}d_{ij}$$

The density $\rho_{i}$ for each skier is defined as the inverse of the average distance to their nearest neighbors:

(7) $$\rho_{i}=\frac{1}{\bar{d_{i}}}=\frac{1}{\frac{1}{k}\sum_{j=1}^{k}d_{ij}}$$

This means that if the average distance between a skier and their neighbors is small, the density is high, and vice versa. The core idea is that the shorter the distance between a skier and their nearest neighbors, the more crowded the area, leading to a higher density. Conversely, the longer the distance to neighbors, the less crowded the area, leading to a lower density.

In the context of skier movement, flow represents the relationship between the density of skiers and their velocity. Using the hydrodynamic relation q=ρv, it is calculated as the product of the density ρ and the velocity *v*, while the local density is determined by averaging over the five nearest skiers.

### 5.2. Velocity vs. Density

As we know, one of the fundamental concepts in crowd dynamics is the relationship between density and velocity, often visualized in the form of a fundamental diagram. In our analysis, we selected four segments from the footage data. These four periods were randomly chosen from the total of eight segments, ensuring that they provide a representative sample for examining the density–velocity relationship.

**Figure 20:**
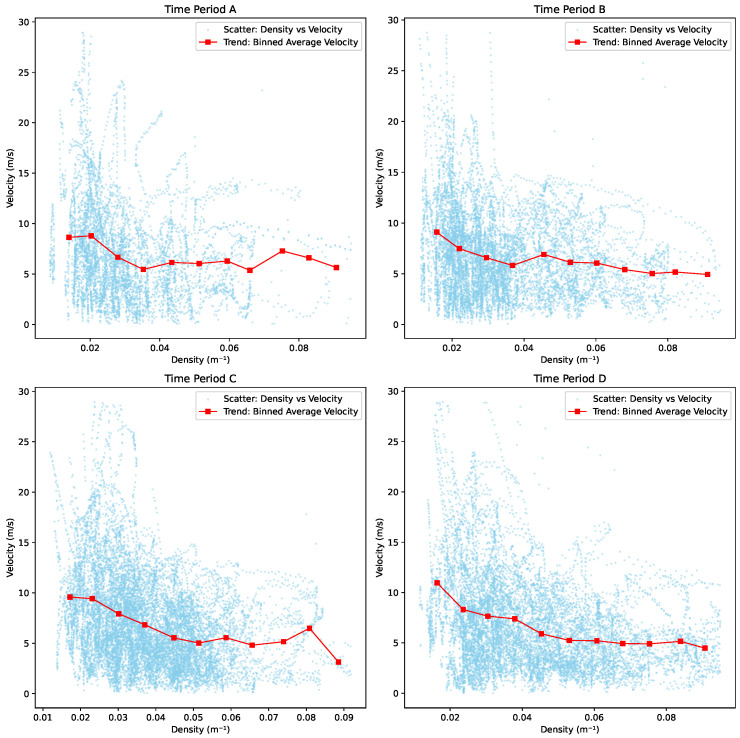
Fundamental diagram: velocity vs. density.

As illustrated in Figure 20, the trends across the four time periods exhibit a similar pattern, with velocity generally decreasing as density increases. In Period A, the downward trend is less pronounced, and the scatter points are the lowest among the four periods. This may explain why there is minimal variation in velocity as density increases during this period. In contrast, Period B shows a more distinct downward trend compared to Period A. For Period C, a notable turning point occurs when density reaches approximately 0.082 m^−1^. After this point, velocity declines sharply as density continues to rise. Period D also exhibits a consistent downward trend; however, the rate of decrease is higher in the density range of 0.02 m^−1^ to 0.04 m^−1^ compared to the range between 0.05 m^−1^ and 0.07 m^−1^. This suggests a variation in how density impacts velocity within different intervals.

**Figure 21:**
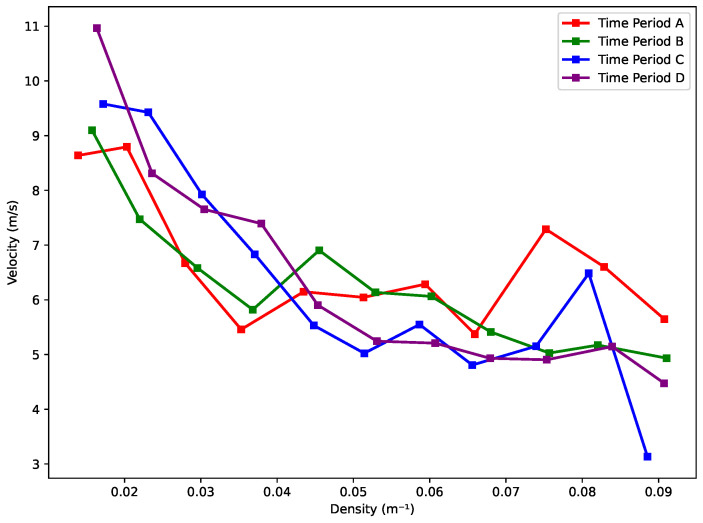
Velocity vs. density trend.

By compiling the line charts for all four periods (Figure 21), a consistent pattern emerges across the different segments. Notably, three out of four periods exhibit a turning point at a density of approximately 0.082 m^−1^. Beyond this critical density, skier velocity decreases significantly, suggesting that increasing density leads to constrained movement and necessitates deceleration to maintain safety. This threshold likely marks a transition from free-flowing to congested conditions on the slope.

In the intermediate density range of 0.04 m^−1^ to 0.082 m^−1^, the velocity–density relationship varies across periods. Period A exhibits minor fluctuations, with two cycles of increase and decrease, followed by a larger rise and subsequent decline. Period B shows an initial small increase, followed by a long, gradual decrease, and a slight rise thereafter. Period C demonstrates alternating downward and upward trends twice. In contrast, Period D presents a more uniform decrease in velocity, with only a very slight increase. These variations suggest that within this density range, skiers have greater flexibility in adjusting their velocities, allowing for intermittent acceleration and deceleration. This indicates that this interval represents a transitional zone where skier movement is less constrained, offering greater adaptability in velocity adjustments. Additionally, the analysis suggests that a comfortable velocity range for skiers falls between 5 m/s and 8 m/s.

### 5.3. Flow vs. Density

**Figure 22:**
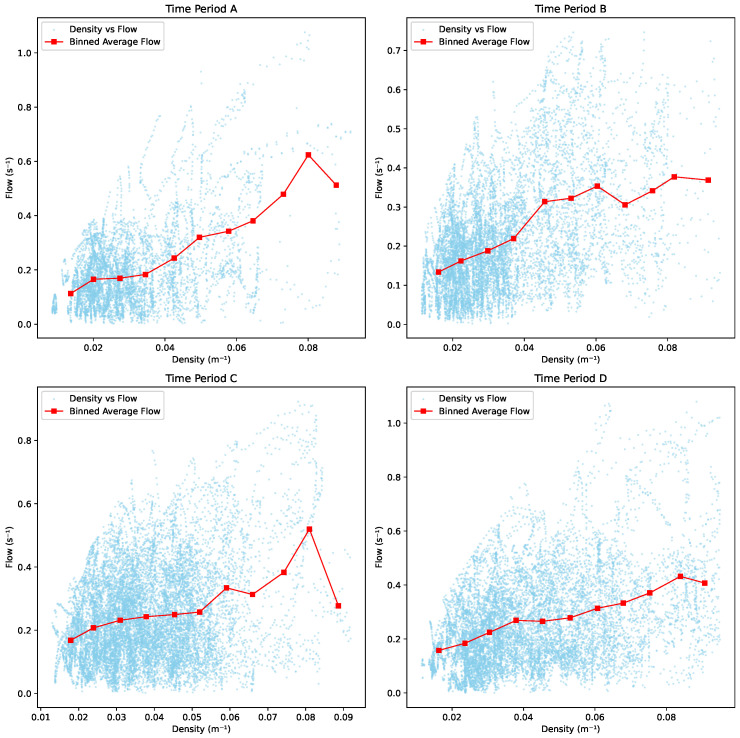
Fundamental diagram: flow vs. density.

As depicted in Figure 22, the flow dynamics across different periods exhibit similar trends. In Period A, the flow generally increases with density, reaching its peak at a density of 0.082 m^−1^, followed by a subsequent decline. In Period B, the flow initially increases gradually with density but begins to decline after reaching a peak at approximately 0.35 s^−1^. A minor fluctuation in flow is observed between densities of 0.06 m^−1^ and 0.082 m^−1^, before the downward trend resumes. Period C displays a clear upward trend in flow, peaking at around 0.53 s^−1^, followed by a decline with only minor fluctuations. Period D shows a steady increase in flow until a density of 0.082 m^−1^ is reached, after which the flow begins to gradually decrease.

**Figure 23:**
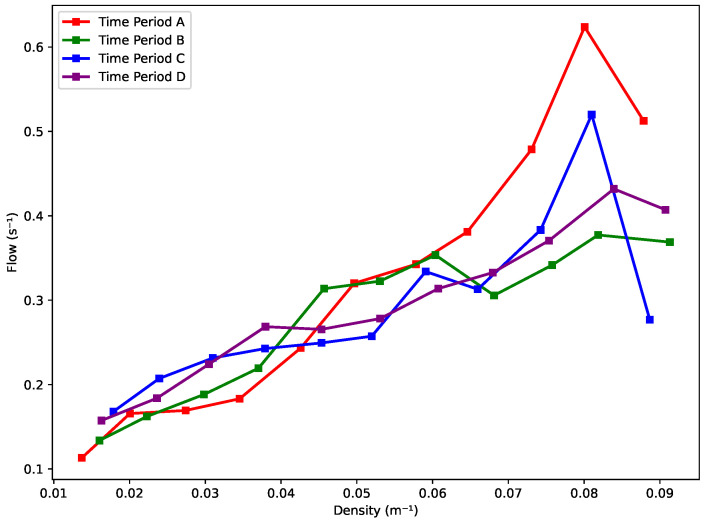
Flow vs. density trend.

From Figure 23, it is evident that an increase in density does not always correspond to an increase in flow. In all four periods, the flow trends begin to decline when the density approaches 0.082 m^−1^. In Period A, the flow experiences two distinct increases as density grows, with the first interval between 0.02 m^−1^ and 0.05 m^−1^, and the second between 0.05 m^−1^ and 0.082 m^−1^. In Period B, the flow increases rapidly until the density reaches 0.046 m^−1^, followed by a slowly increase and a significant decline, and then a subsequent increase before finally decreasing. In Period C, the flow initially rises slowly to 0.26 s^−1^, followed by minor fluctuations, and then quickly peaks when density reaches 0.08 m^−1^. In Period D, the flow steadily increases until it peaks at 0.42 s^−1^, after which it begins to decline.

Interestingly, we do not see a real congested branch in the fundamental diagram where the flow decreases with increasing density. This is similar to the situation found empirically in ant trails [36] and quite different from most other traffic systems.

### 5.4. Velocity vs. Flow

**Figure 24:**
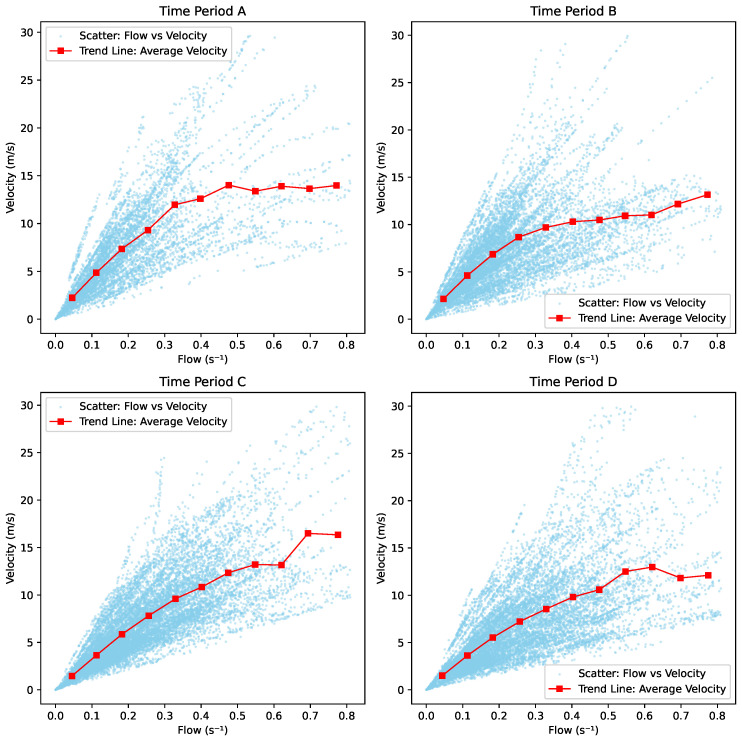
Velocity vs. flow.

As shown in Figure 24, during Period A, the velocity increases steadily to 12 m/s as the flow ranges from 0.1 s^−1^ to 0.5 s^−1^, followed by a slower increase to 14 m/s, with almost no further changes in velocity as the flow continues to grow. In Period B, the flow increases gradually, reaching a velocity of 12 m/s. Period C exhibits two distinct regions: a slow increase in flow until the velocity reaches 12 m/s, followed by a much faster rise, and then transitioning to a horizontal trend. In Period D, a turning point is observed at a flow rate of approximately 0.5 s^−1^, after which the velocity undergoes a slight increase followed by a decline, eventually leveling off at a horizontal line.

**Figure 25:**
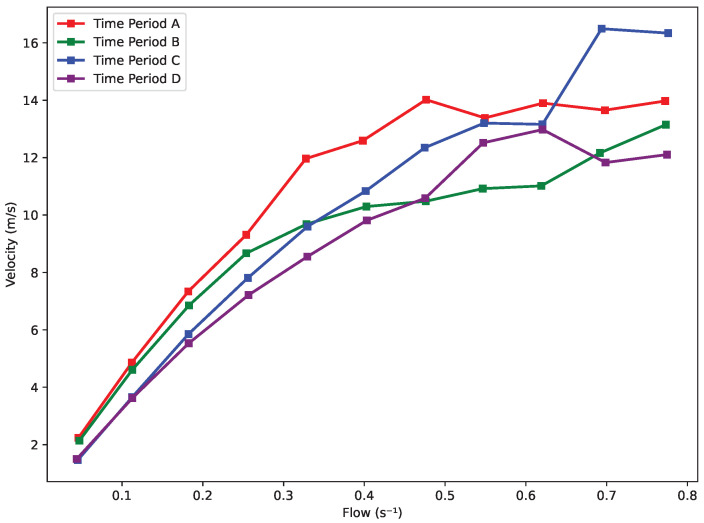
Velocity vs. flow trend.

As observed in Figure 25, all lines exhibit three distinct regions. In the first region, when the flow ranges from 0.1 s^−1^ to 0.5 s^−1^, the velocity increases steadily. In the second region, the velocity alternates between slight rises and declines, with Skier C reaching a peak of approximately 17 m/s. In the third region, the velocity levels off as the flow continues to increase. This pattern indicates the presence of a transition point around 0.5 s^−1^ and 0.7 s^−1^.

### 5.5. Summary

Our study on the fundamental diagram has yielded several key findings. First, we introduced the methodology used to calculate density and flow. From the resulting density vs. velocity diagram, we identified two critical points at 0.04 m^−1^ and 0.082 m^−1^. When the density is below 0.04 m^−1^, velocity decreases as density increases. However, in the range of 0.04 m^−1^ to 0.082 m^−1^, velocity exhibits greater variability, indicating a “comfort zone” where skiers can accelerate or decelerate more freely. Beyond 0.082 m^−1^, velocity decreases rapidly. Similar to mixed traffic flow studies where control strategies such as platoon control and lane management influence fundamental diagram properties [37], our findings suggest that skier interactions shape the density–speed relationship despite the absence of fixed lanes.

For the density vs. flow relationship, we can see that the flow increases steadily as density rises between 0.02 m^−1^ and 0.06 m^−1^. However, in the interval between 0.06 m^−1^ and 0.082 m^−1^, fluctuations begin to appear, suggesting a small oscillating phase before the flow reaches its peak. After 0.082 m^−1^, the flow decreases in all periods. Overall, the shape is more similar to the fundamental diagram observed in ant trails, where no congested phase was found, than those of highway traffic. Unlike road traffic, where well-defined lanes structure vehicle movement, skiing traffic occurs in an open environment where skiers have substantial lateral freedom. As a result, skiing does not strictly follow classical lane-based traffic models. Instead, our results align more closely with pedestrian dynamics in open spaces or ant trails, where self-organized movement naturally gives rise to density–speed–flow relationships.

Additionally, the relationship between velocity and flow shows interesting behavior. We identified two critical flow points at 0.5 s^−1^ and 0.7 s^−1^. Below 0.5 s^−1^, velocity increases slowly. Between 0.5 s^−1^ and 0.7 s^−1^, the velocity would go up and down with a little instability, but when the velocity exceeds 0.7 s^−1^, the flow begins to keep horizontal.

## 6. Conclusions and Discussion

In this article, we considered downhill skiing as a traffic system. We explored several key aspects of skier dynamics, including turning points along trajectories, the minimum distance between skiers, and the fundamental diagram of skiers on the slope. Through this analysis, we identified methods to classify skiers based on their behavior on the slope.

First, we identified three key factors: turning angle, velocity change, and curvature that exhibit significant differences at turning points. Our analysis revealed that conservative skiers tend to make turns with smaller turning angles, maintain greater stability, and exhibit smaller curvature and minimal velocity changes around their turning points. In contrast, aggressive skiers are characterized by more variable turning angles, larger curvature, and greater velocity changes. Furthermore, we observed that skiers’ velocities generally increase as they descend the slope, particularly in the y-direction. Turning points are typically identified when a skier’s velocity in the x-direction approaches zero. This approach provides an effective method for distinguishing skiers’ movement styles by analyzing their turning points and the associated factors.

Next, we explored the minimum distance (MD) between each skier and other agents. By utilizing the MD matrix, we identified each skier’s nearest neighbor and evaluated their typical proximity. Through the analysis of MD changes over time, we found that the typical MD between skiers ranges from 3 to 5 m. For further analysis, we established a threshold of 2 m to define close contact. Based on this criterion, we categorized skiers according to their skiing styles. The results showed that aggressive skiers tend to approach others more frequently and maintain a stable distance, while less skilled skiers demonstrate less consistency in maintaining distance and tend to stay farther from others. This approach offers an effective method for classifying skiers and assessing their skill levels.

Lastly, in our study of skiers’ dynamics on the slope, we determined fundamental diagrams for several periods of footage from various skiing areas and uncovered some key findings. Across the relationships between velocity and density, flow and density, and velocity and flow, we observed a three-region structure. In each region, these relationships exhibit distinct behaviors. Additionally, we found that skiers tend to maintain a “comfortable” velocity range between 5 m/s and 8 m/s. For the density–flow relationship, we identified that while flow generally increases with density, it eventually reaches a saturation point, after which further increases in density lead to diminished flow and some instability. This non-linear relationship underscores the complexity of skier movement and crowd dynamics on the slope. In the velocity–flow relationship, the most harmonious interval occurs before the flow reaches 0.5 s^−1^, during which velocity gradually increases with rising flow.

Compared to mountain bike trajectory studies, which often utilize GPS-based tracking to analyze optimal paths and energy expenditure, our research focuses on skier movement influenced by slope conditions and turning dynamics. Similarly, while pedestrian evacuation models emphasize interpersonal interaction and collision avoidance, skier interactions are largely dictated by velocity differences and terrain adaptability. This distinction underscores the necessity of specialized modeling approaches for skiing dynamics.

Beyond skier behavior, external factors such as terrain and weather conditions can also influence movement patterns. Steeper slopes lead to higher speeds and wider turns, while gentler slopes allow for more controlled movements. Snow quality affects stability—soft snow requires more adjustments, whereas hard-packed snow facilitates smoother trajectories. Additionally, poor visibility may result in more cautious skiing, and strong winds can impact balance and trajectory. Future work could incorporate these factors to develop a more comprehensive model for skiing dynamics and safety improvements.

We hope that our findings offer valuable insights for research and for operators of ski resorts. While this study primarily focuses on the statistical characteristics of skier trajectories, our findings may also have implications for ski resort management and slope safety improvements. The analysis of skier turning behavior and minimum distance distributions provides insights into areas of high skier density, which could inform strategies to enhance safety and optimize infrastructure design. Furthermore, the fundamental diagram analysis highlights how skiing motion exhibits characteristics similar to self-organized pedestrian flow, offering potential applications in dynamic skier flow management.

## Figures and Tables

**Table 1:** Statistics of skier contacts and distances: “minimum distance” is here abbreviated as MD. Additionally, the high contact counts for some skiers may seem surprising. It is important to clarify that for every frame in which the distance between a skier and another agent is less than 2 m, we register one contact count. Therefore, skiers with high contact counts are likely following others closely throughout their journey.

Skier ID	Contact Count	Average MD	MD Std Dev	Minimum of MD
1	22	11.27	6.49	0.95
2	22	6.13	3.18	0.95
3	0	9.30	3.89	4.23
4	4	6.50	3.12	1.96
5	41	9.01	6.80	1.35
6	63	4.74	2.22	1.35
7	84	7.07	6.27	0.71
8	58	4.28	2.90	0.71
9	0	9.82	4.16	2.86
10	0	6.24	1.83	3.27
11	0	7.11	3.02	2.30
12	180	3.71	3.44	0.56
13	180	5.33	4.15	0.56
14	42	6.97	4.12	1.43
15	57	8.90	6.34	1.07
16	34	3.66	1.43	1.61
17	32	5.15	2.22	0.25
18	51	3.65	1.25	0.25
19	127	4.14	3.45	0.06
20	173	2.72	2.27	0.08
21	67	3.70	2.79	0.06
22	44	5.60	4.04	0.48
23	77	2.98	1.31	1.17
24	53	3.71	1.79	0.79
25	0	7.29	2.53	3.58
26	0	8.10	2.23	2.79
27	25	6.52	2.77	1.47
28	25	4.54	2.60	1.47
29	221	2.02	1.55	0.35
30	221	2.99	3.72	0.35
31	26	5.06	2.21	1.41
32	69	6.38	4.19	0.87
33	43	6.72	3.58	0.87
34	30	7.03	3.82	1.14
35	184	2.52	1.38	0.49
36	163	2.68	1.45	0.49
37	0	7.99	3.89	9.68
38	0	8.11	4.45	3.31
39	12	6.84	3.66	1.53
40	35	4.93	2.12	0.92
41	47	4.18	2.55	0.92
42	0	6.34	4.11	3.31
43	0	5.10	2.67	2.67
44	65	4.36	1.65	0.62
45	0	9.43	5.20	3.58
46	0	6.71	2.08	3.85
47	65	5.76	3.91	0.62
48	9	6.15	3.06	1.94
49	9	5.52	1.78	1.94
50	273	2.78	1.82	0.18
51	413	2.32	2.33	0.18
52	0	7.27	2.44	3.76
53	157	4.20	4.38	1.06
54	127	3.62	1.84	0.46
55	227	5.07	4.51	0.46
56	0	7.28	4.22	2.25
57	179	4.12	4.38	0.76
58	14	3.68	3.99	1.95
59	14	4.03	1.95	1.95
60	0	7.22	1.58	5.67

**Table 2:** Cluster feature analysis. Note that all data are the means of the four attributes of the skiers in the cluster.

Cluster	Contact Count	Average MD	MD Std Dev	Minimum of MD
0	14.40	7.95	4.07	2.97
1	36.58	4.97	2.58	1.25
2	201.79	3.44	2.91	0.43

## Data Availability

The data that support the findings of this study are available from the corresponding author upon reasonable request.

## References

[1] Reid R.C., Haugen P., Gilgien M., Kipp R.W., Smith G.A. (2020). Alpine Ski Motion Characteristics in Slalom. Front. Sports Act. Living.

[2] Gilgien M., Spoerri J., Chardonnens J., Kroell J., Limpach P., Mueller E. (2015). Determination of the centre of mass kinematics in alpine skiing using differential global navigation satellite systems. J. Sports Sci..

[3] Supej M. (2008). Differential specific mechanical energy as a quality parameter in racing alpine skiing. J. Appl. Biomech..

[4] Muller E., Bartlett R., Raschner C., Schwameder H., Benko-Bernwick U., Lindinger S. (1998). Comparisons of the ski turn techniques of experienced and intermediate skiers. J. Sports Sci..

[5] Sandbakk S.B., Supej M., Sandbakk O., Holmberg H.C. (2014). Downhill turn techniques and associated physical characteristics in cross-country skiers. Scand. J. Med. Sci. Sports.

[6] Antekolovic L., Cigrovski V., Horgas A. Some Biomechanical Characteristics of Slalom Turn During Race of Elite Alpine Skiers. Proceedings of the 10th International Conference on Kinanthropology: Sport and Quality of Life.

[7] Vaverka F., Vodickova S., Elfmark M. (2012). Kinetic Analysis of Ski Turns Based on Measured Ground Reaction Forces. J. Appl. Biomech..

[8] HIRANO Y., TADA N. (1994). Mechanics of a Turning Snow Ski. Int. J. Mech. Sci..

[9] Hirano Y., Tada N. (1996). Numerical simulation of a turning alpine ski during recreational skiing. Med. Sci. Sports Exerc..

[10] Kondo A., Doki H., Hirose K. (2012). An attempt of a new motion measurement method for alpine ski turns using inertial sensors. Procedia Eng..

[11] Hirose K., Doki H. (2011). A proposal for the motion analysis method of skiing turn by measurement of orientation and gliding trajectory. Procedia Eng..

[12] Thorwartl C., Kroell J., Tschepp A., Holzer H., Teufl W., Stoeggl T. (2022). Validation of a Sensor-Based Dynamic Ski Deflection Measurement in the Lab and Proof-of-Concept Field Investigation. Sensors.

[13] Ruedl G., Kopp M., Burtscher M., Bauer R., Benedetto K. (2013). Causes and Factors Associated with Collisions on Ski Slopes. Sportverletz.-Sportschaden.

[14] Matter P., Ziegler W.J., Holzach P. (1987). Skiing accidents in the past 15 years. J. Sports Sci..

[15] Lochner S.J., Kunz S.N., Fischer F.T., Grove C. (2015). Fatal skiing accidents. Forensic expert opinion at the Institute of Forensic Medicine Munich 2004–2014. Rechtsmedizin.

[16] Ruedl G., Helle K., Tecklenburg K., Schranz A., Fink C., Posch M., Burtscher M. (2015). Impact of Self-Reported Fatigue on ACL Injuries in Alpine Skiing: A Sex Comparison. Sportverletz.-Sportschaden.

[17] Yamazaki J., Gilgien M., Kleiven S., Mcintosh A.S., Nachbauer W., Mueller E., Bere T., Bahr R., Krosshaug T. (2015). Analysis of a Severe Head Injury in World Cup Alpine Skiing. Med. Sci. Sports Exerc..

[18] Treiber M., Germ R., Kesting A. (2015). From drivers to athletes: Modeling and simulating cross-country skiing marathons. Traffic and Granular Flow ’13.

[19] Holleczek T., Troester G. (2012). Particle-based model for skiing traffic. Phys. Rev. E.

[20] Korecki T., Palka D., Was J. (2016). Adaptation of Social Force Model for simulation of downhill skiing. J. Comput. Sci..

[21] Fu Z., Li T., Deng Q., Schadschneider A., Luo L., Ma J. (2021). Effect of turning curvature on the single-file dynamics of pedestrian flow: An experimental study. Phys. A-Stat. Mech. Its Appl..

[22] He Y., Jia F., Kun W., Cao J., Chen S., Wan Y. (2023). Modeling and simulation of lane-changing and collision avoiding autonomous vehicles on superhighways. Phys. A-Stat. Mech. Its Appl..

[23] Cai C., Yao X. (2021). Dynamic analysis and trajectory optimization for the nonlinear ski-skier system. Control Eng. Pract..

[24] Eberle R., Kaps P., Oberguggenberger M. (2019). A multibody simulation study of alpine ski vibrations caused by random slope roughness. J. Sound Vib..

[25] Combinido J.S.L., Lim M.T. (2010). Modeling U-turn traffic flow. Phys. A-Stat. Mech. Its Appl..

[26] Li X., Sun J.Q. (2017). Studies of vehicle lane-changing dynamics and its effect on traffic efficiency, safety and environmental impact. Phys. A-Stat. Mech. Its Appl..

[27] Boltes M., Seyfried A., Steffen B., Schadschneider A., Klingsch W., Schadschneider A., Schreckenberg M. (2010). Automatic Extraction of Pedestrian Trajectories from Video Recordings. Proceedings of the Pedestrian And Evacuation Dynamics 2008.

[28] Boltes M., Seyfried A. (2013). Collecting pedestrian trajectories. Neurocomputing.

[29] Zhang B., Dressler T., Maurer A., Nader M., Schadschneider A. (2024). Simulation of Downhill Skiing Areas. Collect. Dyn..

[30] Delibasic B., Radovanovic S., Jovanovic M., Vukicevic M., Suknovic M. (2017). An Investigation of Human Trajectories in Ski Resorts. Commun. Comput. Inf. Sci..

[31] Yurtsever E., Takeda K., Miyajima C. Traffic Trajectory History and Drive Path Generation Using GPS Data Cloud. Proceedings of the 2015 IEEE Intelligent Vehicles Symposium (IV).

[32] Holst A., Jonasson A. (2013). Classification of movement patterns in skiing. Front. Artif. Intell. Appl..

[33] Gao N., Jin H., Guo J., Ren G., Yang C. (2024). Biodynamic Analysis of Alpine Skiing with a Skier-Ski-Snow Interaction Model. arXiv.

[34] Zhang B., Schadschneider A. (2025). Correcting Ski Resort Trajectories Extracted from Video. Appl. Sci..

[35] Xie C.Z., Tang T.Q., Zhang B.T., Nicolas A. (2023). Adult–child pairs walking down stairs: Empirical analysis and optimal-step-based modeling of a complex pedestrian flow, with an exploration of flow-improvement strategies. J. Stat. Mech. Theory Exp..

[36] John A., Schadschneider A., Chowdhury D., Nishinari K. (2009). Trafficlike Collective Movement of Ants on Trails: Absence of a Jammed Phase. Phys. Rev. Lett..

[37] Peng Y., Liu D., Wu S., Yang X., Wang Y., Zou Y. (2025). Enhancing Mixed Traffic Flow with Platoon Control and Lane Management for Connected and Autonomous Vehicles. Sensors.

